# Novel Treatment of Small-Cell Neuroendocrine of the Vagina

**DOI:** 10.1155/2018/9157036

**Published:** 2018-02-04

**Authors:** Kathryn Kostamo, Mishka Peart, Nathalie McKenzie, Conisha Holloman, S. J. Carlan, Li Ge, John Maksem

**Affiliations:** ^1^Department of Obstetrics and Gynecology, Orlando Regional Healthcare, Orlando, FL, USA; ^2^Gynecologic Oncology, Florida Hospital Medical Group, Maitland, FL, USA; ^3^Department of Pathology, Orlando Regional Healthcare, Orlando, FL, USA

## Abstract

**Background:**

Primary vaginal small-cell neuroendocrine carcinoma is an extremely rare and highly aggressive malignancy. Eighty-five percent of patients die within one year of diagnosis from metastatic disease despite multimodal therapy. Gene expression profiling of tumor tissue may be useful for treatment options for various malignancies.

**Case:**

A 34-year-old nulliparous woman was diagnosed with primary vaginal small-cell neuroendocrine carcinoma. Twenty weeks after the initial visit, she was diagnosed with recurrence and started on chemoradiation based on the results of gene expression profile of tumor tissue. She died 34 months after the initial visit and had a 14-month progression-free survival (PFS).

**Conclusion:**

Gene expression profile of tumor tissue in the management of primary vaginal small-cell neuroendocrine carcinoma may be helpful in extending progression-free survival.

## 1. Introduction

Primary small-cell neuroendocrine carcinoma (SCNEC) of the vagina is a rare tumor with aggressive clinical behavior and poor prognosis despite the current multimodal therapeutic options [1].

The mean age at diagnosis is 59 years, and women typically present with postmenopausal bleeding. Characteristically, this malignancy results in lymphovascular space invasion, and the clinical course is marked by early hematogenous widespread dissemination and early demise [2].

Recently, gene expression testing designed specifically to determine the expression levels of selected biomarkers within tumor samples using quantitative polymerase chain reaction (QPCR) has been offered for certain malignancies including gynecologic phenotypes [3]. Results can be compared with a registry of patients to determine which therapy may be most effective. Whether an MP- (molecular profile-) suggested regimen can improve progression-free survival (PFS) in primary SCNEC of the vagina is not known. We report a case of primary SCNEC of the vagina treated initially with radical surgery and then with combination chemoradiation based on molecular-profiling results after recurrence was documented.

## 2. Case

**Figure 1:**
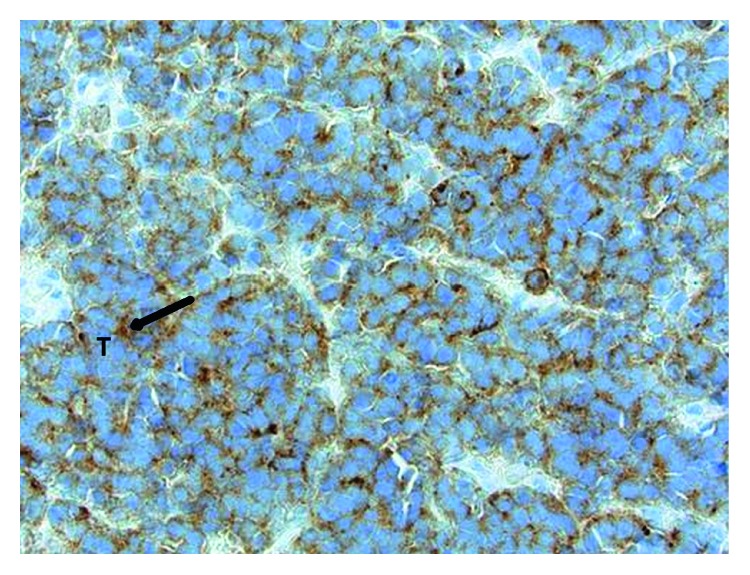
Initial vaginal biopsy of the mass revealing poorly differentiated carcinoma with neuroendocrine features. The tumor cells show cytoplasmic staining with synaptophysin (black arrow), a marker protein for neuroendocrine cells. The staining surrounds the bluish nucleoli of tumor cells (T).

An asymptomatic 34-year-old nulliparous was found to have a 1 × 1 cm, right-sided, firm, nontender vaginal polypoid mass 2 cm from the cervix. A vaginal biopsy of the mass revealed poorly differentiated carcinoma with neuroendocrine features. Immunochemistry demonstrated dot-like staining focally for keratin 20 and diffused weak staining for synaptophysin (Figure 1) Positron emission tomography-computed tomography (PET/CT) done 5 weeks after initial visit showed only a hypermetabolic area localized to the vagina without evidence of regional or distant metastasis.

Eight weeks after the initial visit, she underwent a radical hysterectomy, right salpingo-oophorectomy, left oopexy, partial vaginectomy, and right pelvic lymphadenectomy. The greatest tumor width was 2.3 cm with invasion of deep soft tissue present. Negative vaginal margins were present, and all other findings were benign. Final pathology confirmed stage PT2N0M0 or International Federation of Gynecology and Obstetrics (FIGO) stage II high-grade neuroendocrine tumor of the vagina, Merkel subtype, with negative thyroid transcription factor-1 (TTF-1), CD99, and p63 noted on immunohistochemistry. The patient's tumor was sent for gene expression testing to a Clinical Laboratory Improvement Amendments (CLIA) certified laboratory for chemo- and radiosensitivity profiling for targeted therapy (CerviGENE (PBS78) and Kay's Array (PBS79), OvaGene Oncology, Inc., Irvine, CA). Ribonucleic acid (RNA) was purified from five 10-micron slices of the sample using formalin-fixed paraffin-embedded tissue samples. Expression levels were determined using quantitative PCR. The results showed tumor sensitivity to chemoradiation with improved response to topoisomerase-1 inhibitors and antifolate therapies and a decreased response to platinum agents and gemcitabine. Based on these results, topotecan combined with external beam radiation was presented as a possible treatment course. After extensively counseling the patient on the aggressive nature of her tumor and high likelihood of recurrence, the patient decided to forgo any additional treatments at that time. A surveillance strategy of pelvic exam with Pap smear of vaginal cuff every 3 months and biannual PET/CT scans for 2 years was selected.

**Figure 2:**
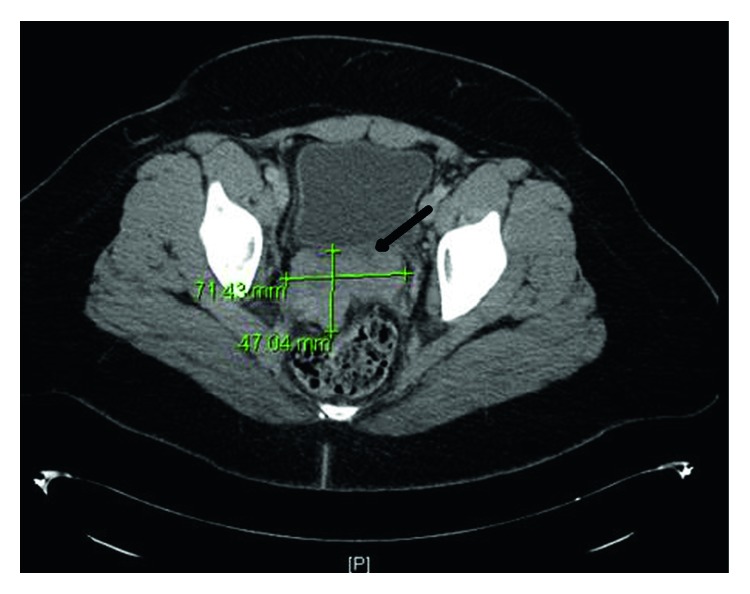
CT of the abdomen and pelvis shows a bulky and irregular pelvic mass measuring 7 × 5 × 6 cm (seen at the arrow in Figure 1).

Twenty weeks after the initial visit, she presented secondary to acute lower abdominal and back pain. CT of the abdomen and pelvis showed a bulky and irregular pelvic mass measuring 7 × 5 × 6 cm (Figure 2) with right obstructive uropathy secondary to ureteral encasement by the pelvic mass. In addition, two separate mesenteric masses, suspicious for metastatic disease, and bilateral external iliac lymphadenopathy were noted. She then agreed to initiate chemotherapy with topotecan, paclitaxel, and bevacizumab for a total of 5 of 6 planned cycles secondary to grade 3 peripheral neuropathy. She was discharged, and 8 months after the initial visit, a magnetic resonance imaging (MRI) demonstrated decreased pelvic lymphadenopathy and pelvic tumor burden with almost complete resolution. The recommendation at this time was for consolidation radiotherapy with whole pelvic eternal beam intensity-modulated radiation therapy (IMRT) in 28 fractions with a total of 50 Grays to the whole pelvis and high-dose-rate (HDR) intracavitary brachytherapy in 3 fractions of 6 Grays. She completed radiotherapy one year after the initial visit without complications, and an MRI of the abdomen and pelvis done at 14 months after the initial visit demonstrated no evidence of new or recurrent disease in the abdomen or pelvis.

Twenty-eight months after the initial visit, she was seen with persistent abdominal pain and vomiting. A CT-guided biopsy of the left retroperitoneal lymph node was positive for metastatic small-cell neuroendocrine carcinoma. Twenty-nine months after the initial visit, she was noted to have brain metastasis, and 33 months after the initial visit, she was started on topotecan, docetaxel, and bevacizumab. She died at 34 months after the initial visit.

A Merkel cell polyomavirus stain performed on the tissue block was negative. The stain was performed postmortem on tissue collected prior to death during the preparation for the case report.

## 3. Discussion

There are three outstanding elements in this case. First, the vaginal location of this malignancy is extremely rare [4] and requires the exclusion of metastasis from other more common sites such as the cervix and the lungs [5]. Only 26 cases have been described prior to this report [6]. Because of this extremely low incidence, no specific treatment guidelines have been established, and most of what is clinically known is derived from occasional single case reports or adopted from that used to approach small-cell carcinoma of the cervix [2].

Second, the initial histopathologic diagnosis of this tumor was as a Merkel cell cancer (MCC) of the vagina. MCC of the vagina is a rare phenotype of aggressive small-cell neuroendocrine tumor, and the diagnosis is extremely difficult because of its rarity and overlapping histology with other tumors [6–8]. It only became clear that it was not an MCC when a postmortem Merkel cell polyomavirus immunostain demonstrated the absence of the virus. The pathogenesis of MCC remains unclear, but the presence of Merkel cell polyomavirus in the tumor genome seems to play a key role, and laboratory confirmation of past viral infection is now possible [9, 10]. The connection between the virus and MCC was not clearly established at the time of the initial diagnosis; consequently, testing for the virus was not part of the original lab analysis. Nonetheless, the therapeutic options were similar for both aggressive recurrent MCC and SCNEC of the vagina.

The third and most important component of this case was the use of molecular profiling to customize treatment. Currently, there is no consensus on the optimal therapy of primary SCNEC of the vagina [5].

Surgical resection or radiotherapy or both may provide increased local control, and chemotherapy is frequently employed as part of the therapeutic regimen. Despite a multimodal approach and using multidisciplinary guidance, prognosis is poor since distant metastasis is the likely outcome. In an effort to improve the response to therapy, gene expression testing that compares the patient's molecular profile to a registry of patients with similar malignancies can be performed. The gene expression testing is designed to specifically determine the expression levels of selected biomarkers within formalin-fixed paraffin-embedded tumor samples. The biomarkers selected for testing are based on extensive literature that strongly supports the gene as a biomarker for a specific drug and for the response to that drug in a specific tumor type. To determine the best likely response to therapeutic options, we used a registry of 98 small-cell cervical cancer patients and tested for 40 gene expression levels. Based on the gene expression levels, the patient was treated with combination chemoradiation starting 20 weeks after her initial visit when recurrence was first documented.

Considering that current therapies for treatment of SCNEC have usually resulted in poor outcomes [11], the fact that the patient had a documented 14-month PFS after starting therapy based on the molecular profiling suggests possibly some benefit. Moreover, vaginal small-cell NETs have a propensity for early widespread dissemination. Eighty-five percent of patients die within one year of diagnosis [5, 12], and survival after diagnosis of recurrent disease is short, typically only 7 months [13]. Our patient's course was consistent with the literature when she developed recurrence 20 weeks after her initial visit. However, her progression-free survival (PFS) extended after 14 months from her first recurrence using the gene expression profile report. Typically, PFS in recurrent gynecologic small-cell neuroendocrine carcinoma of the cervix in treated patients is 7-8 months [14]. She died 34 months after the initial visit.

Despite the fact that most studies investigating molecular profiling of patients' tumors to find potential targets and select treatments for their malignancies are not randomized and do not have controls, most multicentre pilot studies conclude that MP-suggested regimen can be helpful especially in refractory cancers. In summary, comparing these observations to published case reports, it appears that there may be a benefit for adding an MP-suggested regimen to multimodal therapeutic approach for recurrent SCNEC of the vagina.
